# Dronedarone: A New Therapeutic Alternative for Cardiac Arrhythmias

**DOI:** 10.4103/0975-1483.71620

**Published:** 2010

**Authors:** A Upaganlawar, H Gandhi, R Balaraman

**Affiliations:** *Department of Pharmacy, Faculty of Technology and Engineering, MS University of Baroda, Kalabhavan, Vadodara – 390 001, Gujarat, India*

**Figure 1:**
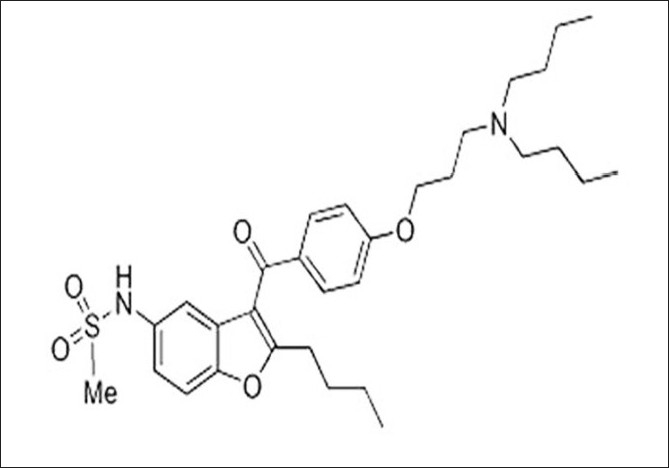
Structure of dronedarone IUPAC name: N-(2-butyl-3-(p-(3-(dibutylamino) propoxy)benzoyl)-5(benzoyfuranyl methanesulfonamide; Chemical class: Benzofuran derivative

Sir,

As a new therapeutic agent for cardiac arrhythmias, dronedarone (Multaq ^®^), a drug from Sanofi-Aventis, was approved by the FDA on July 2, 2009.[1] It is now available in the market in the form of tablets having 400 mg of the active ingredient. For patients requiring drug therapy or electroshock therapy to retain their normal rhythms, dronedarone is a useful option. However, treatment with dronedarone was associated with an increased likelihood in deaths due to heart failure.[2] Hence, the FDA did not approve dronedarone without a warning for causing deaths in patients with heart failure.[3] Figure 1 shows the structure, the International Union of Pure and Applied Chemists (IUPAC) name, and chemical class of dronedarone.

For getting FDA approval, the manufacturer of dronedarone was compelled to add a black box warning to the package insert. The warning is as follows: ‘WARNING: HEART FAILURE: MULTAQ is contraindicated in patients with NYHA class IV heart failure, or NYHA class II-III heart failure with a recent decompensation requiring hospitalization or referral to a specialized heart failure clinic.’

The pharmacokinetics of dronedarone is dictated by its lipophilicity. Dronedarone is less lipophilic than amiodarone and consequently it has a much smaller volume of distribution. It has an elimination t_1/2_ of 24 hours as compared to amiodarone’s half-life of a few weeks; this is also attributed to the lower lipophilicity of dronedarone.[4]

In the trials in atrial fibrillation (EURIS, 2007, and ADONIS, 2007), significantly better maintenance of the sinus rhythm was observed with dronedarone than with placebo. In the short term, lung and thyroid function remained unaffected.[5] Conversely, in an another study (ANDROMEDA, 2007), dronedarone doubled the death rate as compared to placebo and the trial had to be stopped prematurely.[2] Later, it was found that ANDROMEDA enrolled patients with moderate to severe congestive heart failure and had a relatively sicker patient population.

The typical adverse effects observed with dronedarone include nausea, diarrhea, rash, and creatinine elevation.[6]
